# Diagnostics of Cognitive Function in Women of Menopausal Period at Level of Primary Health Care

**DOI:** 10.5195/cajgh.2014.183

**Published:** 2014-12-12

**Authors:** N. Zhilgeldina, T. Ulykbassova

**Affiliations:** Department of Ob/Gyn, National Research Center for Maternal and Child Health, Astana, Kazakhstan

**Keywords:** menopause, cognitive function, psychological function, women’s health

## Abstract

**Introduction:**

In the medical community, there is no consensus on whether or not climacteric changes are pathologic and require treatment. One of the main problems related to menopause is misperception of menopause; consequently, there is no consensus on treatments for psychological dysfunction and cognitive deficits in menopausal women. Timely diagnosis and adequate treatment of psychological disorders and cognitive dysfunction are imperative and complicated. The purpose of this study was to evaluate physician perceptions of cognitive and psychological deficits in menopausal women in outpatient settings.

**Methods:**

215 obstetricians-gynecologists working in out-patient services were surveyed using a multiple choice questionnaire assessing perceptions and knowledge of menopausal transition.

**Results:**

Of total respondents, 42.0% ± 2.5 of physicians found it hard to define menopausal period, and 67.5% ± 3.2 could not give a clear definition of hormone replacement therapy. On the question “cognitive function includes…,” 62.5% ± 2.1 of physicians selected “memory,” 32.3% ± 1.8 selected attention, 77.5% ± 3.2 selected mood and/or imagination, 37.4% selected intellect, 36.3% ± 3.1 of respondents selected character traits, and 6.2% ± 1.7 selected speech. Regarding the question “how do you study memory status function?” it was estimated that 71.2% ± 2.5 of study participants have studied the memory only on the basis of subjective complaints, and none of the respondents (100%) have ever used neuropsychological tests.

**Conclusion:**

The survey allows us to ascertain that primary medical care services lack the ability to appropriately recognize and diagnose cognitive deficits in women of menopausal age. Based on these data, we can assume that proper mental care is not provided. Thus, the study indicates a need to create training programs for general practitioners and other specialists (cardiologists, neurologists, and endocrinologists) to fulfill this need. Implementation of a standard of care, testing, and treatment of cognitive and psychological function, such as the use of neuropsychological tests and questionnaires, in an out-patient setting for menopausal women would improve the quality of life during a woman’s transition period.

